# Nicht sexuell erworbene genitale Ulzerationen: Eine Fallserie zum Lipschütz‐Ulkus

**DOI:** 10.1111/ddg.15815_g

**Published:** 2025-11-14

**Authors:** Julian Steininger, Michael Eckert, Sophia Lehr, Susanne Abraham, Stefan Beissert, Claudia Günther

**Affiliations:** ^1^ Klinik und Poliklinik für Dermatologie Universitätsklinikum Carl Gustav Carus Technische Universität Dresden; ^2^ Praxis für Gynäkologie und Geburtshilfe, Vilsbiburg; ^3^ Universitäts‐Hautklinik Universitätsklinik Tübingen Eberhard Karls Universität Tübingen

Sehr geehrte Herausgeber,

Das Ulcus vulvae acutum beziehungsweise Lipschütz‐Ulkus ist eine seltene, nicht sexuell übertragbare Erkrankung, die durch das akute Auftreten schmerzhafter Ulzerationen der Vulva und Vagina gekennzeichnet ist. Das Lipschütz‐Ulkus stellt eine wichtige Differenzialdiagnose zur Abgrenzung gegenüber sexuell und nicht sexuell übertragbaren Infektionen, Autoimmunerkrankungen wie Morbus Behçet oder Morbus Crohn, medikamenteninduzierten Reaktionen sowie lokalisierten Manifestationen sonstiger systemischer Erkrankungen dar.^1^ Der Krankheitsverlauf ist typischerweise selbstlimitierend, weshalb die Therapie primär symptomatisch mit Analgetika erfolgt.

In diesem Bericht werden zwei Fälle junger Patientinnen beschrieben, die sich mit extrem schmerzhaften vulvären Ulzerationen vorstellten.

Die erste Patientin war eine 16‐jährige Jugendliche, die sich mit seit zwei Tagen bestehenden Schmerzen im Bereich der Vulva vorstellte. Anamnestisch habe eine viertägige Prodromalphase mit Fieber bis 40°C, Halsschmerzen, Husten sowie submandibulärer Lymphknotenschwellung bestanden. Klinisch zeigten sich ödematöse Erytheme sowie ein 2 × 1 cm großes, hämorrhagisches Ulkus mit fibrinösen Rändern am rechten Labium majus sowie ein 1 cm großes, fibrinbelegtes Ulkus an der anterioren Vaginalwand (Abbildung 1a). Ähnliche Beschwerden in der Vergangenheit sowie sexuelle Aktivität wurden jeweils verneint. Die Patientin berichtete jedoch über wiederholt aufgetretene orale Ulzerationen in der Kindheit.

**ABBILDUNG 1:**
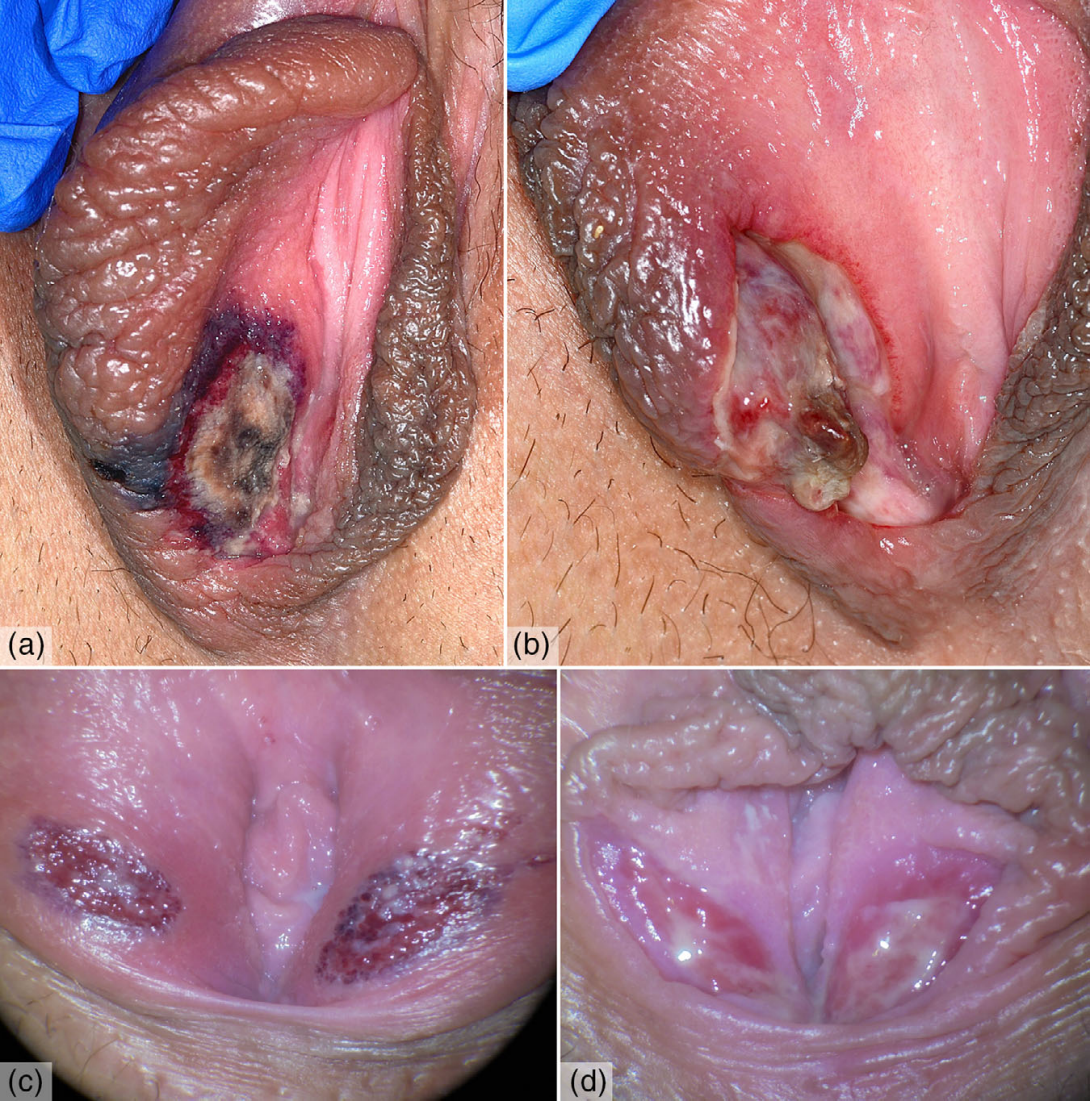
Klinische Präsentation von zwei Patientinnen mit Ulcus vulvae acutum. (a) Schmerzhaftes, hämorrhagisches und fibrinöses Ulkus im Bereich des rechten Labium majus bei Aufnahme. (b) Frühphase der Heilung mit fibrinösem Umbau 6 Tage später. (c) Symmetrische, erythematöse und peripher bläulich‐livide Ulzerationen an beiden Labia minora bei Aufnahme (Originalvergrößerung x 7,5). (d) Granulationsgewebe nach 4 Wochen (Originalvergrößerung x 7,5).

Es erfolgte eine umfangreiche Diagnostik zur Abklärung potenzieller viraler, bakterieller sowie autoimmunologischer Ursachen. Die serologische Testung auf Zytomegalievirus, Epstein‐Barr‐Virus, Parvovirus B19 und Syphilis sowie der PCR‐Nachweis von Mykoplasmen waren jeweils unauffällig. Ebenso zeigten Abstriche der Läsionen keinen Nachweis von Herpes‐simplex‐Virus Typ 1 oder 2. Daraufhin wurde die initiale Aciclovir‐Therapie beendet und eine antibiotische Prophylaxe mit Cefuroxim eingeleitet. Auch die Testergebnisse relevanter Autoantikörper, einschließlich ANA, waren negativ. Die histologische Analyse ergab eine gemischtzellige Entzündung ohne Anzeichen einer Vaskulitis, was zur Diagnose eines Lipschütz‐Ulkus führte. Die Ulzerationen zeigten bereits nach wenigen Tagen einen fibrinösen Umbau (Abbildung 1b) und heilten innerhalb von sechs Wochen vollständig und ohne Narbenbildung ab.

Patientin 2 war eine 21‐jährige Frau, die sich mit symmetrischen, erythematösen und peripher bläulich‐livide verfärbten Ulzera an beiden Labia minora vorstellte (Abbildung 1c). Dem Auftreten der Läsionen seien anamnestisch keine Infektionszeichen oder eine Verschlechterung des Allgemeinzustands vorausgegangen. Der letzte Geschlechtsverkehr der Patientin habe eine Woche vor der Vorstellung stattgefunden. Analog zu Patientin 1 waren die viralen und bakteriellen Tests negativ, und die histologische Untersuchung zeigte eine unspezifische Entzündung. Die Therapie erfolgte mit topischen Glukokortikoiden, Lidocain und Analgetika. Nach 4 Wochen entwickelte sich reizloses Granulationsgewebe (Abbildung 1d). Bei der regelmäßigen Nachkontrolle 3 Monate später war eine vollständige und komplikationslose Abheilung feststellbar.

Das Lipschütz‐Ulkus, erstmals 1912 beschrieben, manifestiert sich durch einzelne oder multiple Vulvaläsionen, die häufig von Fieber und Lymphadenopathie begleitet werden.^2^ Die Ulzerationen weisen typischerweise einen gelblich‐grauen, fibrinösen Grund mit erythematösen Rändern auf und sind mit starken Schmerzen assoziiert, die die Mobilität und Miktion beeinträchtigen können.^3^ Obwohl Lipschütz‐Ulzerationen in jedem Alter auftreten können, werden sie am häufigsten bei sexuell inaktiven jungen Frauen beobachtet.^4^, ^5^, ^6^ Zudem kann eine Assoziation mit viralen und bakteriellen Infektionen, insbesondere akuten Epstein‐Barr‐Virus‐Infektionen, bestehen.^4^, ^5^, ^6^, ^7^, ^8^, ^9^ Folglich gehen dem Auftreten der Ulzera oft grippeähnliche oder pharyngeale Symptome voraus.

Die genaue Pathogenese ist weiterhin unklar. Die Histologie ist typischerweise unspezifisch^6^, ^10^ und dient primär dem Ausschluss von Vaskulitis oder Malignität. Das Lipschütz‐Ulkus ist selbstlimitierend und heilt in der Regel innerhalb von 10 Tagen bis 6 Wochen ohne Narbenbildung ab. Die Standardtherapie ist daher supportiv und umfasst Antiseptika, Analgetika sowie Maßnahmen zur Förderung der Wundheilung. Obwohl in der Vergangenheit häufig systemische Kortikosteroide zur Beschleunigung des Heilungsprozesses eingesetzt wurden, deuten neuere Daten darauf hin, dass dieser Ansatz keinen signifikanten Nutzen erbringt.^9^

Retrospektive Daten von 110 Frauen mit Genitalulzera zeigten, dass bei 30 % ein Lipschütz‐Ulkus diagnostiziert wurde. Weiterhin berichtete ein Drittel dieser Frauen über mindestens eine frühere ähnliche Episode.^6^ Dies unterstreicht die Bedeutung der Berücksichtigung des Lipschütz‐Ulkus in der Differenzialdiagnose genitaler Ulzerationen, insbesondere bei sexuell inaktiven jungen Frauen, um unnötige Behandlungen zu vermeiden und ein adäquates Management zu gewährleisten.

Es ist wichtig zu berücksichtigen, dass serologische Tests – insbesondere in sehr frühen Infektionsstadien – aufgrund des diagnostischen Fensters, in dem Antikörper möglicherweise noch nicht nachweisbar sind, Einschränkungen aufweisen können. Daher sollten bei unklaren Befunden wiederholte Testungen oder alternative diagnostische Verfahren in Erwägung gezogen werden. In den vorliegenden Fällen unterstützte die initiale umfassende Diagnostik in Verbindung mit dem klinischen Bild und den histologischen Befunden eindeutig die Diagnose eines Lipschütz‐Ulkus, wodurch keine erneuten Tests notwendig waren.

## DANKSAGUNG

Open access Veröffentlichung ermöglicht und organisiert durch Projekt DEAL.

## INTERESSENKONFLIKT

Keiner.
